# Nerve, Muscle, and Synaptogenesis

**DOI:** 10.3390/cells8111448

**Published:** 2019-11-16

**Authors:** Lauren Eric Swenarchuk

**Affiliations:** 580 Christie Street, Suite 914, Toronto, ON M6G 3E3, Canada; l.swenarchuk@utoronto.ca; Tel.: +1-416-658-7747

**Keywords:** synapse, agrin, MuSK, Lrp4, neuromuscular junction

## Abstract

The vertebrate skeletal neuromuscular junction (NMJ) has long served as a model system for studying synapse structure, function, and development. Over the last several decades, a neuron-specific isoform of agrin, a heparan sulfate proteoglycan, has been identified as playing a central role in synapse formation at all vertebrate skeletal neuromuscular synapses. While agrin was initially postulated to be the inductive molecule that initiates synaptogenesis, this model has been modified in response to work showing that postsynaptic differentiation can develop in the absence of innervation, and that synapses can form in transgenic mice in which the agrin gene is ablated. In place of a unitary mechanism for neuromuscular synapse formation, studies in both mice and zebrafish have led to the proposal that two mechanisms mediate synaptogenesis, with some synapses being induced by nerve contact while others involve the incorporation of prepatterned postsynaptic structures. Moreover, the current model also proposes that agrin can serve two functions, to induce synaptogenesis and to stabilize new synapses, once these are formed. This review examines the evidence for these propositions, and concludes that it remains possible that a single molecular mechanism mediates synaptogenesis at all NMJs, and that agrin acts as a stabilizer, while its role as inducer is open to question. Moreover, if agrin does not act to initiate synaptogenesis, it follows that as yet uncharacterized molecular interactions are required to play this essential inductive role. Several alternatives to agrin for this function are suggested, including focal pericellular proteolysis and integrin signaling, but all require experimental validation.

## 1. Introduction

How is the vertebrate skeletal neuromuscular junction (NMJ) made? Specifically, what molecular interaction initiates synaptogenesis when nerve and muscle meet? Despite almost half a century of work, a detailed picture has yet to emerge, and it is unclear as to whether a single process or multiple mechanisms are involved. This review does not aspire to resolve matters, but aims rather to outline the basic arguments at issue.

The NMJ acts as a simple relay, and unlike many central synapses, has no capacity for modifying its performance. However, this very simplicity, coupled with its large size and relative accessibility, has made it a favored object of study for elucidating basic principles of synapse structure and physiology that are relevant not only to its own functioning, but to that of central synapses as well. This expectation has certainly proved to be true historically, as studies of the NMJ gave us the first ever concept of a membrane receptor (“receptive substance”) [1], convincing evidence for the chemical nature of synaptic transmission [2] and, ultimately, key physiological and ultrastructural evidence for the quantal mechanism of transmitter release [3].

Throughout millions of years of vertebrate evolution, the NMJ has remained relatively constant, with no evidence that selective pressures have led to alterations in molecular components of the synaptic machinery or to any evolutionary improvement in function. For example, few if any mammals can match frogs in their ability to leap from a standing start [4]. In studies of NMJ development, this structural and functional equivalence has been reflected in an implicit assumption, invoking Occam’s razor, that the molecular mechanisms underlying its formation have also been conserved throughout the vertebrate realm.

## 2. The Agrin Hypothesis

### 2.1. Historical Development of the Model

Early investigations in adult mammals and frogs demonstrated that transplanted nerves could form synapses on any part of the muscle surface. These studies usually involved inactivation of existing synapses, either by denervation [5] or treatment with botulinum toxin [6], although this inactivation was not always required [7]. In the late 1970s, these findings were extended through the integration of two lines of work. Using the *Xenopus* in-vitro system, Anderson and Cohen [8] found that synapses formed anywhere on the surface of embryonic muscle, leading to the dispersal of existing clusters of acetylcholine receptors (AChRs) and their lateral migration to re-assemble along the path of nerve-muscle contact; this conclusion was supported by other in-vitro studies in chick [9] and mammals [10], and was consistent with the earlier studies in adult muscle. Meanwhile, in U.J. McMahan’s laboratory, work involving ablation of frog nerve and muscle demonstrated that regenerating nerves grew back to pre-existing sites of synaptic differentiation in the muscle basal lamina [11], and that AChR clusters developed under these same sites in the membrane of regenerating muscle [12]. Thus, the basal lamina was implicated in two functions, providing termination signals for the nerve and AChR aggregation signals for the muscle. In subsequent work, the larger size of the muscle, permitting a relatively straightforward examination of the distribution of key synaptic markers, led to a principal focus on the latter capability.

These findings led to the formulation of the “agrin hypothesis” to account for postsynaptic differentiation [13]. According to this account, a protein, named agrin for its ability to induce AChR aggregation, is deposited by the developing nerve in the muscle basal lamina, not only inducing the aggregation of AChRs under the nerve during embryogenesis, but also providing inductive signals to regenerating muscle. In the literature, this function is typically characterized as “organizing” the postsynaptic membrane, with agrin designated as “organizer”, whose action results in the localization of existing components to the point of nerve-muscle contact. It should be noted, however, that this inductive process may include the new synthesis of synaptic components as well, such as basal lamina constituents [14]. In the present discussion the term “inducer” is preferred to include this aspect, while explicitly referring to the initial interaction between nerve and muscle membranes.

Over a decade of studies led to agrin’s isolation and cloning, a body of work which is especially notable for the underlying assumption of a unitary molecular mechanism, one that was meant to be applied across the span of vertebrate classes and skeletal fiber types. While this point was rarely if ever emphasized explicitly, it was implicit in the potpourri of vertebrate species involved. Thus the biochemical isolation of agrin employed tissue from a ray, assays for activity were carried out in avian muscle cultures [15], and immunocytochemical localization was done in frog, ray and rat [16,17]. In addition, at a time when cloning a gene was a significant undertaking, agrin was cloned no fewer than three times, employing libraries from rat [18], chick [19], and ray [20]. In parallel with these efforts, the molecule which ultimately proved to be the key postsynaptic transducer of agrin-mediated signaling, a receptor tyrosine kinase, was cloned first from *Torpedo* [21], and subsequently from human, rat, and mouse libraries, and named MuSK (muscle specific kinase) [22].

Ultimately, this led to gene ablation studies with transgenic mice. Not only were mice lacking agrin unable to make synapses [23], but the cause of this defect was localized to the absence of one neuron-specific isoform possessing an exon coding for only eight amino acids [24]. In parallel, the role of MuSK was also delineated [25], and evidence was also adduced supporting the involvement of another postsynaptic protein as an essential cofactor [26]; this was later identified as low density lipoprotein receptor-related protein 4 (Lrp4); [27,28,29]. The agrin/MuSK findings were treated as a signal event, meriting comment in publications as diverse as Cell [30] and the New York Times [31]. As we entered the new millennium, the basic problem thus seemed solved, and the agrin hypothesis reigned supreme [32].

### 2.2. Challenge to the Model

Acceptance of the standard version of the agrin hypothesis proved short-lived, however, as a number of studies, whether in vivo, focused on the rodent diaphragm [33,34,35], or in vitro, using mammalian myotubes [36], showed that significant postsynaptic differentiation could proceed in the absence of nerve. Moreover, these studies provided evidence that the role of agrin might be to stabilize existing AChR aggregates in the face of a neurally derived dispersive agent [34,35]. This work culminated with evidence provided by two different groups, one of which, including J.R. Sanes and colleagues [37], had carried through the key agrin knockouts discussed above [23,24], and another, working independently [38]. Using double knockouts of agrin and choline acetyl transferase, the enzyme responsible for ACh synthesis, these authors found that synapses now formed. Furthermore, both groups adduced evidence in support of a mechanism whereby agrin is required to maintain synaptic AChR clusters, which in its absence would be dispersed, owing to an inhibitory mechanism mediated by the neuronal release of ACh in the region of the developing synapse. This dispersal was attributed in part to AChR endocytosis, as well as to lateral diffusion through the membrane [37]. In these transgenics, given the ablation of choline acetyl transferase expression and consequent lack of ACh, agrin was no longer essential, as it was not required to antagonize any ACh-mediated dispersion.

Research into the mechanism mediating this dispersal by ACh has included differing research emphases, as some studies have concentrated on AChRs at the synapse [37], while others focus on receptors that were not contacted by a nerve [38]. It remains possible, however, that the molecular mechanisms underlying the dispersion of both synaptic and nonsynaptic receptors may be related, in that distant AChRs, like those at the synapse, are associated with specialized extracellular matrices [39,40], and these links must be severed to permit lateral migration of AChRs toward the synapse [8,14]. In any event, a full account of these phenomena remains to be elaborated; to date the activities of several postsynaptic enzymes have been implicated in AChR dispersion, including Cdk5 [38], calpain [41], and caspase-3 [42].

### 2.3. Revised Model for Agrin

The formation of synapses in mice lacking the agrin gene clearly called for a revision of its proposed mode of action. Rather than being the key synaptic organizer, agrin was assigned a role as stabilizing agent, similar to that already proposed in the interpretation of the experiments where postsynaptic differentiation was shown to occur in the absence of nerve [34,35,43]. Specifically, Misgeld et al. [37] summarized their results as follows: “In terms of agrin, we believe that in addition to its role in clustering AChRs, which has been demonstrated in vitro, it also acts to antagonize the effect of ACh. Thus, it is an ‘antideclustering’ agent, instead of or in addition to being a clustering factor.”

Here, Misgeld et al., having just shown that synaptogenesis could proceed in agrin’s absence *in vivo*, still remained unwilling to abandon an inductive role completely. Now, however, they based their conclusions for this capacity purely on in vitro data, rather than on the earlier knockouts that had seemed so compelling. Drawing on two decades of in vitro and in vivo studies, they posed the fundamental issue examined in the present review: does agrin act only as a stabilizer or does it both induce new synapses as well as stabilize them. Given the history of the field, it is of particular note that these authors, whose transgenic studies were crucial in establishing the idea that agrin is an inducer, accepted that this concept might have to be jettisoned altogether.

The overall state of matters was soon addressed in a major review by Sanes and colleagues [44]; the essence of this account is summarized in their title, evoking both Milton [45] and T.S. Kuhn [46]: *“Assembly of the postsynaptic membrane at the neuromuscular junction: paradigm lost.”* There were three principal aspects of the accepted account which required alteration: (i) the evidence for muscle’s ability to generate postsynaptic differentiation in the absence of nerve; (ii) the conclusion that neuregulin-1 was not involved in the induction of synaptogenesis; and (iii) the abandonment of agrin’s straightforward role as the inducer of synaptogenesis. Of these three issues, the most important feature of the “lost paradigm” was the last, the need to reconsider the role of agrin, since this meant that we no longer had a unifying concept, formulated at the molecular level, that could account for the initiation of synapse formation at all vertebrate NMJs.

## 3. Synapse Formation: Two Mechanisms—Agrin and Prepatterning

The verdict rendered in “paradigm lost” is striking, both in terms of its incisiveness and the fact that it originated from one of the leading groups investigating the NMJ. The logic was clear enough, however, and the two reports that synapses could form in the absence of agrin [37,38] were soon joined by a third study in which agrin’s ablation was accompanied by a second genetic transformation, leading to elevated postsynaptic concentrations of MuSK. In this last study, the mice survived, albeit runted, for several weeks [47]. It might have been expected that the modified view of agrin’s action would foster a search for an alternative that could account for the induction of postsynaptic differentiation in agrin’s absence. Surprisingly, this has not happened, and in this respect the contrast with another candidate inducer, neuregulin-1, is notable.

As reviewed by Kummer et al. [44], a number of studies had implicated neuregulin-1 as having an important role, but two transgenic studies, in which synapses continued to form in the absence of either neuregulin-1′s receptors [48], or neuregulin-1 itself [49], essentially refuted this concept, although neuregulin-1 does play key roles in peripheral nervous system function, especially through actions on glia [50], as well as involvement in regulation of AChR synthesis [51,52]. With respect to agrin, however, many reports have simply ignored the findings that synapses can form in its absence. It is not uncommon for later papers, be they introductions of studies [53,54] or reviews [55,56,57], to characterize agrin’s role in terms of its traditional function as an organizer that promotes AChR clustering, with no reference to an alternative mode of action, and without discussing the concerns of Misgeld et al. [37] and Kummer et al. [44].

Instead, in the years following, focus shifted to the role of pre-existing postsynaptic differentiation, termed “prepatterning”. The findings of pre-existing postsynaptic differentiation in rodent muscles were extended by work in zebrafish, where it was reported that some synapses were induced *de novo*, while others incorporated pre-existing AChR clusters [58,59]. Together with the earlier studies of prepatterning, this has led to the conclusion that two mechanisms mediate synaptogenesis at NMJs, one with prepatterning and one without [60,61]. This assumption is made explicit in a Cell SnapShot, generally considered to provide an authoritative perspective, which asserts that “the final cohort of synapses on the muscle fiber includes those recognized by motor axons and those induced by motor axons” [61]. It should be noted, however, that there is an implicit bias toward the set of synapses that are recognized, as opposed to induced, in the general introductory statement that “Muscles are prepatterned … prior to and independent of innervation”; overall, the revised view of agrin as stabilizer is given prominence in the SnapShot. Regarding those synapses that are induced, there exists only the statement that “agrin can also induce new postsynaptic specializations” [61]. No other inductive mechanism is proposed for the cases where prepatterning is missing, such as the in vivo transplantation experiments [5,6,7], and the various in vitro studies where synapses formed anywhere on the muscle surface [8,9,10].

In any event, instead of a straightforward single mechanism for synapse formation and for the role of agrin, the model now posits synaptogenesis as proceeding by two mechanisms and agrin having two functions. While it is not unusual for cellular processes to reflect the operation of redundant mechanisms [62], the question remains as to whether this provides a satisfactory account for the induction of postsynaptic differentiation, the subject of “paradigm lost”. The following sections will analyze this model with respect to two questions: (i) whether it is reasonable to conclude that these two types of synapse formation proceed through a common underlying molecular mechanism, and (ii) whether the available evidence supports two roles for agrin. The conclusions, while provisional and dependent on further investigations for confirmation, are affirmative for the first question, and negative for the second, as it is argued that the available evidence favors agrin’s action as a stabilizer, while its inductive role is open to serious question. A final section suggests alternative inductive mechanisms, which, taken together with a role for agrin as a stabilizing factor, may restore the concept of a unitary mechanism for the initiation of postsynaptic differentiation at all vertebrate NMJs.

Presynaptic differentiation will not be treated, as current conclusions regarding presynaptic differentiation appear more definitive. Evidence has been adduced to implicate all three members of the agrin/MuSK/Lrp4 complex as contributing to induction of presynaptic differentiation [63,64,65] with particularly strong evidence in favor of Lrp4 [65,66]; similarly, despite the importance of Schwann cells for the functioning and maintenance of the NMJ [67,68], their involvement comes at a later stage than the first inductive interactions between nerve and muscle, and accordingly will not be discussed.

### 3.1. Postsynaptic Prepatterning and Synapse Formation

Postsynaptic prepatterning, the aggregation of AChRs in the region where synapses form prior to innervation, provides the substrate for one of the two proposed mechanisms of synapse formation, neuronal incorporation of pre-existing postsynaptic differentiation. Prepatterning was first reported by Braithwaite and Harris [69], who found that ablation of innervation by injection of ß-bungarotoxin still left AChRs assembled in the center of the rat diaphragm. Very similar findings were reported by Yang et al. [33] in a mutant lacking the enzyme topoisomerase-ß, and in transgenics lacking either the transcription factor HB9 or neuronal agrin; prepatterning was, however found to be dependent on MuSK [34,35]. Moreover, in cultured mammalian muscle, elaborate postsynaptic structures form in the absence of nerve [36]. Follow-up studies have been especially informative in detailing the molecular interactions involving agrin, MuSK, and Lrp4, and, in the clinical sphere, the relevance of autoimmune responses to these proteins to the pathophysiology of myasthenia gravis [53,70,71].

This work has yielded two surprises. First, in mammals it has been established that Lrp4 rather than agrin plays the central role in the activation of MuSK, although neuronally supplied agrin does play an essential supportive function, acting as an allosteric regulator of the Lrp4-MuSK interaction, by binding to the extracellular N-terminal region of Lrp4 [29,54,72,73,74,75]. In addition, in zebrafish the activation of MuSK in forming the prepattern is mediated through its binding to Wnt, not Lrp4, although Lrp4 is still required for synapse formation [76,77].

In rodent muscles such as the diaphragm, prepatterned AChR clusters are restricted to a narrow band in the center of the fiber, toward which the nerve terminals grow [34,35,47]. Furthermore, studies in zebrafish have provided examples where the nerve incorporates these pre-existing structures into new synapses [58,59]. At present, however, the number of muscles which have been examined for prepatterning is relatively small. It remains to be seen whether prepatterning plays any role in such large fast muscles as the frog sartorius that lack a defined central band of synapses, or in tonic fibers, that are multiply innervated [78,79].

As to whether prepatterning is essential for synaptogenesis, early reviews were especially cautious, pointing out the studies, outlined in Section 2.1, that synapse formation proceeds in its absence, while suggesting that the prepattern establishes a preferred region for the termination of the final nerve branches [80]. This concept was supported in a later study involving MuSK over-expression, which resulted in the destruction of the prepattern, and axons wandering randomly over the muscle surface [47]. Overall, the evidence appears strong that prepatterning, where it occurs, can direct the final terminating branches on the muscle surface [65,66,76].

#### Refinement of the Prepattern: Implications for A Unitary Molecular Mechanism

The question remains as to whether the appropriation of pre-existing structures by the incoming nerve involves different molecular mechanisms from the case where these structures are absent. In short, how are the synapses, “those recognized by motor axons” [61], actually formed? From first principles, we might reasonably assume that if the incorporation of existing structures is a different process from the induction of a novel postsynaptic apparatus, then different macromolecules on the nerve and muscle surfaces would be expected to mediate the respective interactions between the two cells. It should be noted, however, that the existing prepattern of AChRs is known to be refined by nerve contact, since the final geometry of the mammalian synapse is modified in response to innervation [34,43,44,81]. To date the mechanisms mediating this rearrangement have yet to be examined, raising the question: is this process any different, at the molecular level, from the induced migration of distant AChRs [8], with the exception that the receptors are now, thanks to prepatterning, close at hand?

Several studies suggest that it is not. If different mechanisms were involved in synaptogenesis mediated by incorporation of a prepattern, as opposed to de novo generation of the postsynaptic apparatus, we might anticipate that disrupting the prepattern would have direct impacts on synapse induction. However, such interference does not prevent synapse formation, even in the very preparations where prepatterning has been most intensively studied. For example, in the mouse diaphragm, prepatterning was disrupted in muscle fibers lacking the dihydropyridine receptor essential for excitation-contraction coupling; nevertheless, functional NMJs were formed [82]. Similar results were seen in mice whose muscles expressed elevated levels of MuSK; destruction of the prepattern was accompanied by motor neurons wandering over the entire muscle surface, but functional synapses still developed [47]. Moreover, detailed experimental manipulation of Wnt signaling in zebrafish was found to eliminate prepatterning, but synapse formation still occurred; these authors concluded that prepatterning of AChR is dispensable for the induction of synaptogenesis in this system, even though it plays a central role in restricting the pathfinding of growth cones [76]; see Section 4.2.

The most parsimonious interpretation of these investigations is that the two modes of synapse formation proposed by Sanes and Yamagata [60] and by Burden [61] both reflect an underlying unity of molecular mechanism, expressed in the three general cases examined: (i) in the simplest case, where there is no prepattern, as in the early experimental studies in vitro [8,9,10], and in vivo [5,6,7], postsynaptic structures are induced at the point of cell-cell contact; (ii) if prepatterning is present, the same inductive interactions involved in de novo synaptogenesis mediate the final remodeling of postsynaptic components so as to align prepatterned AChRs with the terminal nerve branches; and (iii) when normal prepatterning is abolished experimentally, so that there are no longer any pre-existing AChR clusters to be re-aligned, the muscle simply uses the same molecular mechanisms to aggregate AChRs *de novo*.

Taken together, these studies lead to two general conclusions. First, whatever assistance prepatterning may provide toward direction of nerve terminal growth, it is not essential for the development of postsynaptic differentiation, the focus of the “lost paradigm”. Second, the fundamental assumption underlying the agrin hypothesis, that a single molecular mechanism mediates postsynaptic differentiation at all vertebrate skeletal NMJs, whether or not prepatterning is present, continues to be a viable proposal. It now remains to analyze whether the original formulation of this concept can be sustained, namely that the secretion of neuronal agrin is the key inductive step.

### 3.2. Agrin—Stabilizer, Inducer, or Both?

In principle, both of the two actions proposed for agrin, induction of new synapses and stabilization of existing ones, are vital, and the loss of either one could account for the lethality of the first agrin knockouts [23,24]. In their discussion of these actions, Misgeld et al. [37] were unwilling to abandon the concept of an inductive role for agrin, even as they provided evidence for a stabilizing function. These possibilities, stabilizer and inducer, are here assessed in turn, drawing not only on the transgenic approaches which have predominated in recent studies, but also on earlier findings from the amphibian culture system.

#### 3.2.1. Agrin as Stabilizer

Acting as an “antideclustering” stabilizing agent, agrin could in principle either be deposited together with an inductive stimulus, or at some time later, after AChR clustering has begun. Since Misgeld et al. [37] proposed this view of agrin’s role, there have been two data sets that might be interpreted as a challenge to the concept. The first was the double transgenic where agrin was ablated and MuSK expression in muscle was simultaneously elevated. In this study motor nerve growth over the muscle surface was aberrantly profuse, but the agrin-less mice, although runted, survived for several weeks, implying a general level of functionality of the NMJs [47]. Thus, even in the face of continued ACh release, which should have led to rapid dispersal of synaptic AChR aggregates with agrin absent [37,38], synapses had formed and were clearly stable for an extended time. Since MuSK over-expression was known to promote MuSK dimerization and consequent MuSK kinase activity, allowing for activation in the absence of agrin [83], persistence of synapses was attributed to MuSK self-activation, owing to its elevated concentration [47]. Although this mechanism has not been explicitly confirmed, it can in principle compensate for the stabilizing action that would normally be provided by the neuronal agrin isoform, and the concept of agrin as stabilizer is accordingly preserved.

The interpretation of MuSK self-activation has been supported by the case of a second double transgenic where agrin was ablated and the mice survived; in this study, the accompanying genetic modification was the elevation of another postsynaptic protein, Downstream of kinases-7 (Dok-7), an activator of MuSK [84]. Once again functional NMJs formed, with the mice surviving for up to two months; moreover, biochemical analyses confirmed that postsynaptic MuSK activity was in fact significantly increased, reflecting the increased synthesis of Dok-7. Ultimately, however, the synapses disintegrated. Thus it appears that elevation of MuSK activity can obviate the need for neuronal agrin over the time period required for induction and consolidation of postsynaptic differentiation, even though over the longer term the system cannot be maintained. Remaining to be explained, however, is the mechanism by which the interaction of nerve and muscle was initiated in the absence of agrin.

#### 3.2.2. Agrin as Inducer

The classic agrin hypothesis was especially attractive in that a single factor was assumed both to initiate a complex process and also to differentiate motor neurons from other nerve types. Thus specificity [85] was in principle accounted for, as it was assumed that motor neurons are specialized for providing neuronal agrin [16,17], even though other studies indicated that sensory neurons do apparently transcribe the gene [86]. Close examination of the evidentiary basis for an inductive action of agrin, however, suggests that it is less strongly supported than is often averred.

##### Early Studies: Issues of Interpretation

Even at the time of its formulation, the agrin hypothesis in practice held a special position, being the only proposal with any general currency, effectively constituting a default hypothesis. In this context a number of nonphysiological approaches, including bath application [87] and postsynaptic expression from injected transgenes [88,89], gave results that were consistent with the agrin hypothesis, and accordingly were taken as being as supportive of it. Ultimately these conclusions appeared to be validated with the agrin knockouts [23,24] and studies with nerve-muscle cultures [90]. It is safe to say, however, that such interpretations would have been subject to more scrutiny if it had been known that synapse formation could proceed in animals lacking the agrin gene. Simple logic would have called for discriminating between inductive and stabilizing roles for agrin, or at least, as in the case of Misgeld et al. [37], making a case for both.

The key element in confirming agrin as an inducing agent is the question of time, meaning that to be the key inducer it must be deposited at an early stage of nerve-muscle contact. To establish this rigorously requires examination of synapse induction as it occurs during nerve-muscle interaction. In this context, we should note one fact that may lead to over-interpretation of many experimental approaches that do not observe this condition; namely, the apparent promiscuity of the muscle surface in its response to exogenous stimuli. For example, two nonphysiological examples that can lead to AChR clustering include local electric fields [91,92], and uncoated polystyrene beads [93]. Thus such approaches as local fixation onto culture surfaces [94,95] are open to the possibility that the muscle membrane is responding to a signal that is not the same as occurs during synaptogenesis. In fact, this caveat may even apply to the original finding that AChR clustering occurs at specialized basal lamina sites of regenerating muscle [12] despite the ultimate productivity of this approach in identifying agrin as a key actor in synapse formation.

In any event, the several reports [37,38,47,84] that mice lacking the agrin gene could form synapses present an obvious challenge that must be answered. In two cases [37,38] one could argue that the completeness of the inductive process was suspect, since the lack of ACh, given the concomitant knockout of choline acetyl transferase, precluded functional analysis of the outcome. The other cases, however, in which either MuSK or Dok-7 was elevated while agrin was ablated [47,84], are more definitive, in that the mice survived for many weeks, so that functional synapses clearly formed. In addition, in one of these reports postsynaptic differentiation could not have proceeded through the incorporation of existing prepatterned structures, since prepatterning was also obliterated [47]. Given that induction by neuronal agrin was obviously precluded, some other process mediated the initiation of synaptogenesis, and what this might be remains a mystery.

There is one major caveat, however, to applying any inferences to NMJ formation more generally, given the nature of the double transgenics in question. Specifically, the elevation in activity of the key postsynaptic transducing molecule can reasonably be taken as such an aberrant circumstance as to preclude using such transgenics to draw definitive conclusions pertaining to wild-type mice. To answer this objection properly requires a context in which agrin is missing at the key inductive juncture, even though there are no major distortions in gene expression of key participating molecules. Fortunately, this condition is satisfied by studies in the amphibian in vitro system, especially when viewed in the light of transgenic data showing that agrin’s action is critically dependent on its binding to laminin in the basal lamina.

##### Agrin in the Amphibian In Vitro System

To establish biological relevance of a potential inductive stimulus, the challenge is not just to demonstrate that it is capable of inducing synaptic differentiation, but that it actually does so in a physiological context, and at the required time. In particular, a candidate inductive process, such as the secretion of neuronal agrin, clearly must occur early in the sequence of events that ultimately generates a synapse. To date the preparation which remains the most closely examined step-by-step, during the process of synapse induction, is the original in vitro system with embryonic cells from *Xenopus laevis* [8]. In addition to facilitating the assessment of the timing of events during synaptogenesis, this culture system is of additional interest in that, like the in vivo nerve transplantation experiments in adult muscle [5,6,7], synaptogenesis proceeds in the absence of prepatterning. In the scheme of Sanes and Yamagata [60] and Burden [61], this is clearly a case of induction of a new synapse, rather than incorporation of existing postsynaptic structures. Accordingly, if agrin acts as an inducer in such cases, as argued in the Cell SnapShot [61], this role should be evident here.

In this preparation it is noteworthy that synaptic differentiation does not occur at an interface with a culture substrate, but rather between nerve and muscle membranes, in essence replicating the in vivo situation. The only difference from the in vivo case is that synapses form without nerve termination; nonetheless they show all the expected features of a differentiated contact by physiological, immunocytochemical, and ultrastructural criteria [96,97]. Using a pulse-chase protocol to stain AChRs first with labeled α-bungaroxin, then following with unlabeled toxin after nerve addition, AChR aggregation under the nerve was shown to include receptors that were stained before nerve addition. These were interpreted as having migrated laterally through the muscle membrane [8], although endocytosis and re-insertion of labeled AChRs may also have contributed to their ultimate localization at the synapse [98]. This aggregation of AChRs was subsequently shown to proceed in parallel with a series of defined stages in the development of a specialized synaptic basal lamina [14,97].

These studies form the backdrop for assessing the role of agrin during synapse formation in this system. The work is almost entirely observational, but taken together with detailed functional analyses of the agrin molecule, provides three related lines of evidence that all point towards agrin as acting to stabilize, rather than induce. These include (i) the role of agrin as a basal lamina constituent, viewed in the context of basal lamina remodeling; (ii) the time course of agrin deposition during synaptogenesis in living cultures; and (iii) the relationship of agrin to stabilization of AChR aggregates at the synapse following denervation.

##### Neuronal Agrin as Basal Lamina Constituent: Implications for Function

Agrin is a large heparan sulfate proteoglycan, approximately 400 Kd, of which half is composed of heparan sulfate side chains [99]; the final protein is subject to differential splicing of the mRNA transcript, but for the purposes of defining its functional role as stabilizer or inducer we are only concerned with the N- and C-terminal regions. First, as discussed in Section 3.1, Lrp4 and MuSK interact directly, with agrin acting to potentiate this interaction by binding to Lrp4 [29,54,72,73,74]. This binding to Lrp4 is effected through the C-terminal region of agrin containing the key 8-amino acid insert that is essential for synapse formation [24]. Elucidating the nature of this interaction has been the major focus of functional studies [56,73], inasmuch as it ultimately leads to downstream postsynaptic interactions.

In contrast, the function of the N-terminal region has been much less examined, but one dedicated study has provided key information of direct relevance to the question of agrin’s role in synaptogenesis. Burgess et al. [100] demonstrated that there are two principle N-terminal agrin variants, isoforms with an extended 150 amino acid sequence, termed LN, and those without, termed SN. While SN-agrin mediates integration into cell membranes throughout the nervous system [100], LN-agrin has a more restricted distribution, with a particular concentration in motor neurons. Moreover, agrin’s incorporation into the muscle basal lamina is a property of the LN-isoforms [100,101,102]. Based on in vitro studies with chick nerve and muscle, as well as transfected mammalian (Cos-7) cells, this longer N-terminal binds to the γ1 chain found in most laminin heterotrimers [103], including those characteristic of the specialized synaptic basal lamina [104,105,106].

Of central importance for the present discussion, this binding is critical for synaptogenesis, as transgenic mice lacking only the N-terminal extension were as deficient in NMJ formation as mice lacking all forms of agrin [100]. This deficiency is unlikely to reflect interference with interactions with Lrp4, since tissue extracts from mice lacking the N-terminal extension retained AChR clustering activity on cultured myotubes [100]. It is most likely therefore, that during synaptogenesis neuronal agrin is not simply secreted onto the muscle surface in a freely diffusible form; instead its C-terminal is presented to Lrp4 while its N-terminal is bound to laminin in the extracellular matrix.

**Figure 1:**
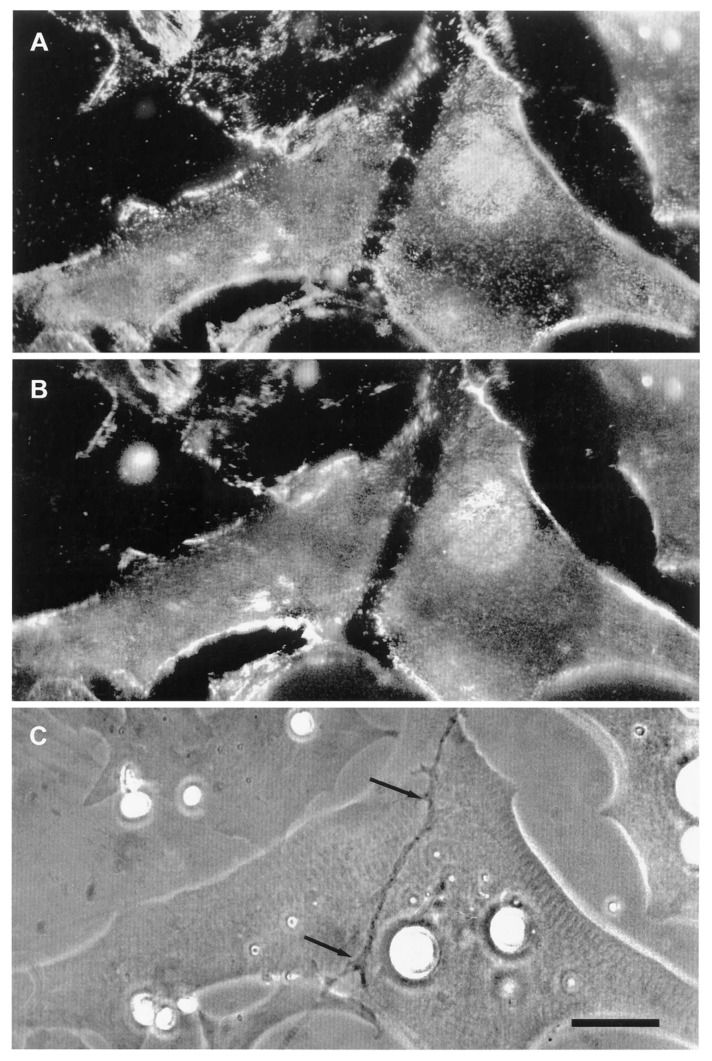
Nerve-induced removal of basal lamina constituents prior to synapse formation. Living nerve-muscle cultures of *Xenopus laevis* were examined approximately one day after the addition of nerve. Two constituent proteins of the muscle basal lamina, a heparan sulfate proteoglycan (HSPG) and laminin, (**A** and **B**, respectively), were stained with monoclonal antibodies labeled with contrasting fluorochromes. Both show a dramatic loss of fluorescence intensity along the path of the nerve, shown in the phase contrast image (**C**). Note that the width of reduced fluorescence in A and B is several-fold greater than the width of the nerve itself. This reduced fluorescence is seen only at those nerve-muscle contacts that go on to make synapses, a 1:1 correlation suggesting that localized pericellular proteolysis of basal lamina constituents may contribute to the transmission of inductive signals between nerve and muscle. Scale: 20 µm. Reproduced with permission from Anderson et al., Mol. Cell. Biol. 1996;16:4972-84. [107]

**Figure 2:**
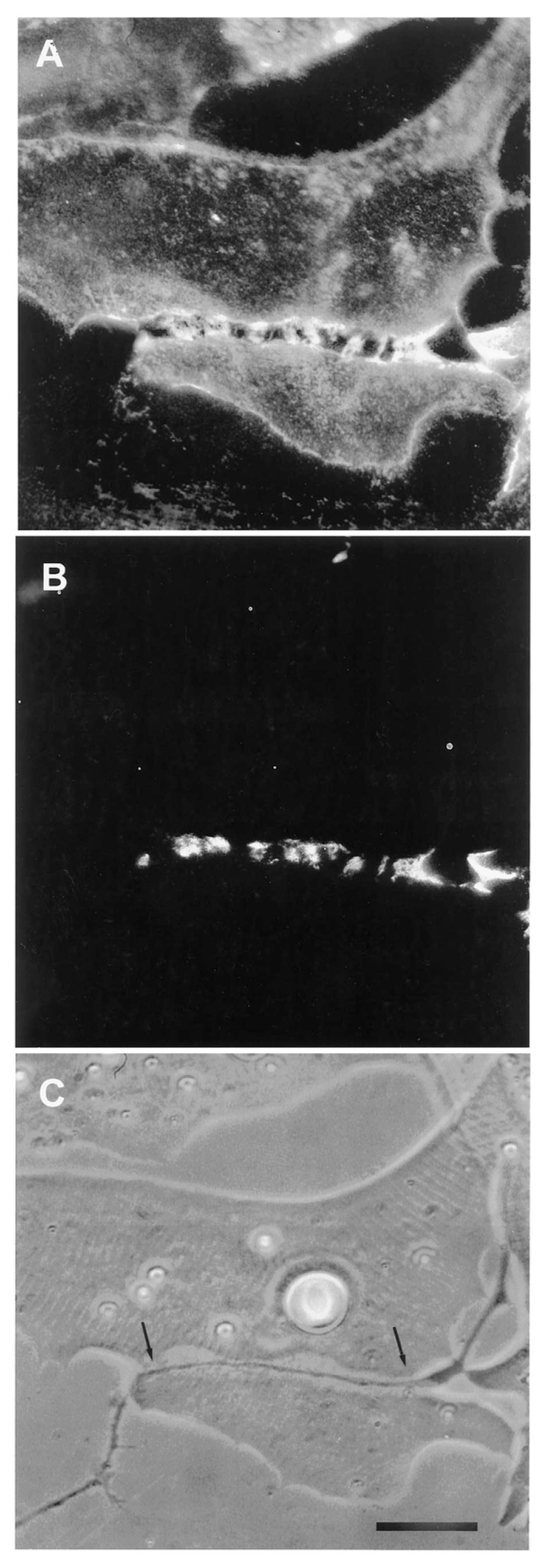
Deposition of a specialized basal lamina parallels synaptic aggregation of acetylcholine receptors in the muscle membrane. (**A**) In this living *Xenopus* culture, examined after several days of nerve-muscle co-culture, laminin, stained with a monoclonal antibody, is beginning to fill in the region where it had formerly been removed along the path of nerve-muscle contact, shown in the phase reference image (**C**) This newly deposited laminin is at a higher site-density than is present elsewhere on the muscle cell. In favorable preparations the entire synaptic zone will be filled in. (**B**) Acetylcholine receptors (AChRs), stained with fluorescent α-bungaroxin, conjugated to a contrasting fluorochrome, have aggregated in the muscle membrane along the path of cell-cell contact, co-localizing with the synaptic laminin deposits seen in A. AChR staining is not evident elsewhere on the muscle surface. Scale: 20 µm. Reproduced with permission from Anderson et al., Mol. Cell. Biol. 1996;16:4972-84. [107]

The relevance of this finding for agrin function becomes clear when we examine the sequence of events during synaptogenesis in the amphibian culture system. Here, the first indication that a given nerve-muscle contact will form a synapse is the removal of existing basal lamina constituents along the path of the nerve. These constituents include the basal lamina heparan sulfate proteoglycan (HSPG) and, importantly, laminin [107]; (see Figure 1). Both molecules, derived from the muscle [108,109], are then re-deposited at high concentration in the synaptic basal lamina [107]; (see Figure 2), a local specialization that frog NMJs share with those of mammals [99,110]. Meanwhile AChR aggregates accumulate in the adjacent muscle membrane [14,107]; (see Figure 2).

Taken together with the findings that agrin is presented in a laminin-bound form, this sequence of removal and re-deposition implies that the incorporation of neuronal agrin into the synaptic cleft follows an earlier inductive process, one that involves basal lamina remodeling. At the earliest stages of nerve-muscle interaction, the removal of laminin from the synaptic cleft precludes the incorporation of agrin into the matrix. Only after laminin is re-deposited can this incorporation proceed, thereby allowing the interaction of agrin with Lrp4. Whether the development of a specialized synaptic basal lamina follows the same time course in other vertebrate species remains to be established, but here in the amphibian in vitro case, where the result is a clearly defined synapse, the sequence provides evidence that agrin deposition occurs relatively late, consistent with agrin’s playing a stabilizing role, rather than acting as an inducer.

It might be thought that drawing inferences from such disparate data, involving as they do in vivo transgenic studies [100], electron microscopic studies of the interacting macromolecules [102], in vitro binding experiments with expression plasmids [103], and in vitro formation of living synapses [107], is forcing the issue. In reality, however, the conclusions depend on only one basic assumption, namely that the molecular interactions between laminin, agrin and Lrp4 are conserved from *Xenopus* to mammals and birds. While ultimately requiring confirmation using *Xenopus*-derived macromolecules, this assumption is reasonable, akin to that which underlay the various studies leading to the identification of agrin: given similarity of organelle structure and function, there is no reason to posit differences at the molecular level.

Finally, the amphibian culture system comprises a second instance in which NMJs are induced without the involvement of either prepatterning or agrin. The first, described in mice by Kim and Burden [47] (Section 3.2.2), eliminated both as a result of the transgenic modifications. In the amphibian cultures, which lack prepatterning, agrin’s absence at the early inductive phase is implicit, given the very early removal of its basal lamina binding partner, laminin [107]. Both cases, therefore, preclude invoking a fallback position for initiating postsynaptic differentiation, whereby either agrin or postsynaptic prepatterning can be absent, since the other’s presence will suffice. In these examples both are absent, and postsynaptic differentiation still proceeds; the straightforward implication is that some other mechanism mediates its induction.

##### Time Course of Agrin Deposition during Synaptogenesis in Living Cultures

**Figure 3:**
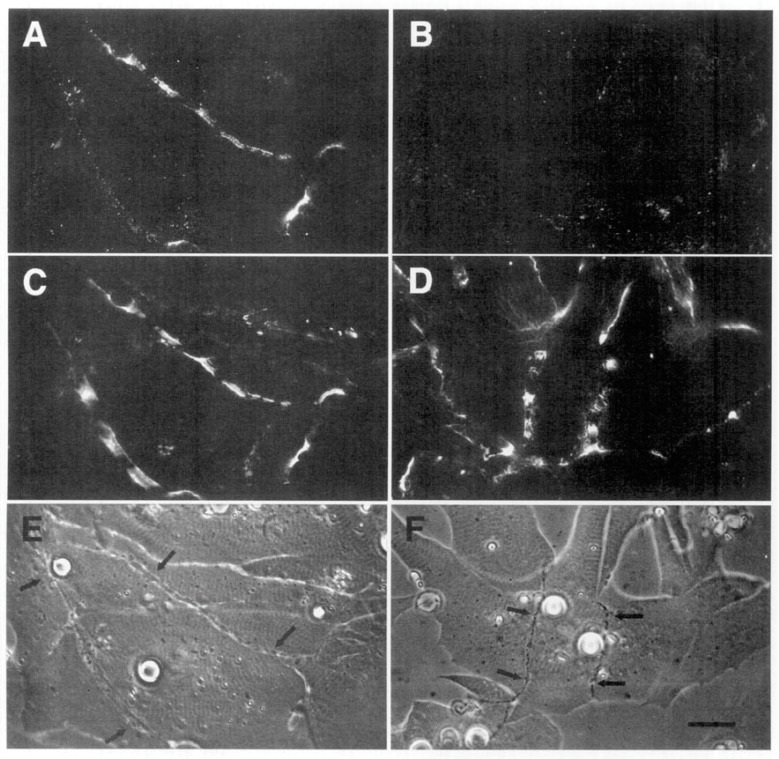
Variation in agrin deposition at different neuromuscular junctions, despite their accessibility to staining with antibodies to the basal lamina HSPG. (**A**,**B**) After several days of nerve-muscle co-culture, staining with an antibody to agrin is marked in upper nerve-muscle contact of panel **A**, corresponding to the nerve running from 11 o’clock to 4 o’clock in the phase reference image (arrows in **E**), while staining is much fainter in the second contact, corresponding to the nerve running from 10 o’clock to 6 o’clock in **E**. In panel B, however, neither of the two nerve-muscle contacts corresponding to the arrows in the phase reference image (**F**) shows any sign of agrin deposition. (**C**,**D**) The basal lamina HSPG, stained with an antibody conjugated to a contrasting fluorochrome, is brightly stained at all four nerve-muscle contacts, in contrast to the variability in staining of agrin. Scale: 20 µm. Reproduced with permission from Anderson et al., Dev. Biol. 1995;170:1-20. [112]

The second line of evidence in support of a stabilizing role for agrin comes from direct observation of its deposition during synapse formation. This question of timing was examined in two studies in the amphibian culture preparation, both carried out in the early 1990s, prior to the agrin knockouts. Cohen and Godfrey [111], examining synapses on the upper muscle surface, found an excellent correlation between the appearance of agrin and concomitant postsynaptic differentiation. However, these results were challenged on technical grounds by Anderson et al. [112], who found that the protocols employed in the earlier study could not discriminate between agrin deposits derived from nerve-muscle contact and those secreted into the culture medium by surrounding epithelial cells. When Anderson et al. examined synapses occurring on the underside of the muscle, they did not find a consistent correlation between synaptogenesis and agrin deposition, even though a strict correlation was observed for deposition of other basal lamina constituents. Moreover, they found that agrin deposition, when it occurred, usually lagged behind other synaptic markers, as shown in the examples of Figure 3.

Here, four different nerve-muscle contacts are visible in the phase contrast reference images (E and F). In panels A and B, all stages of agrin deposition are evident, ranging from absence to high concentration at the synapse. Note that in panel A there is significant agrin deposition corresponding to the upper nerve of panel E, while for the contact in the lower left, there is very little. Meanwhile, in panel B, there is virtually no sign of agrin whatever for either of the nerves visible in F. In contrast, the basal lamina marker, in this case HSPG (panels C and D), is present in high concentration for all four contacts. Had it been possible to employ a third fluorochrome, it would have been evident that AChR aggregation under the nerve, the classic marker for synaptic differentiation, had already occurred [97,107]; see Figure 2 above.

The most straightforward interpretation of these images is that they are essentially snapshots taken of a dynamic process during which the various nerves have contacted the myocytes at different times, with the intensity of agrin staining dependent on the length of time that the two cells have been interacting. In each of the nerve-muscle contacts, basal lamina remodeling has already resulted in heavy deposition of HSPG. The contrasting variability in agrin staining implies that agrin deposition comes relatively late in the sequence of events, after HSPG deposition and AChR aggregation, rather than at the early time point expected for an inducer/organizer. Accordingly, Anderson et al. concluded that the developmental sequence is most consistent with agrin’s acting to stabilize new synapses rather than to induce their formation [112], in effect presaging proposals made in the following decade [43,44,113].

##### Behavior of AChR Clusters after Denervation

The third line of evidence for agrin as a stabilizing factor in these cultures derives from studies of the response to denervation. Anderson et al. [97] reported that when denervation was carried out in the first day after the synapse formed, synaptic AChR clusters invariably disappeared. For technical reasons all the experiments were carried out with curare in the medium; it is required in order to block the effects of large spontaneous miniature end-plate potentials that otherwise induce contractures of the myocyte, leading to its death through tearing off the culture substratum [96]. Unlike the studies of the mammalian situation, where the dispersive effect of neurally-released ACh, reacting with AChRs, had to be counteracted by agrin [37,38], here in the amphibian cultures the AChRs were already blocked, precluding any action of ACh. Even so, the synaptic AChR accumulations were inherently unstable at the earliest stages of synaptogenesis, and the presence of an intact nerve was required to prevent their loss. Similar results were reported by Kuromi and Kidokoro [114], who also examined older nerve-muscle contacts, and found that after three days of co-culture, approximately half the synaptic AChR clusters remained intact after denervation, suggesting that a process of stabilization was taking hold at later times.

**Figure 4:**
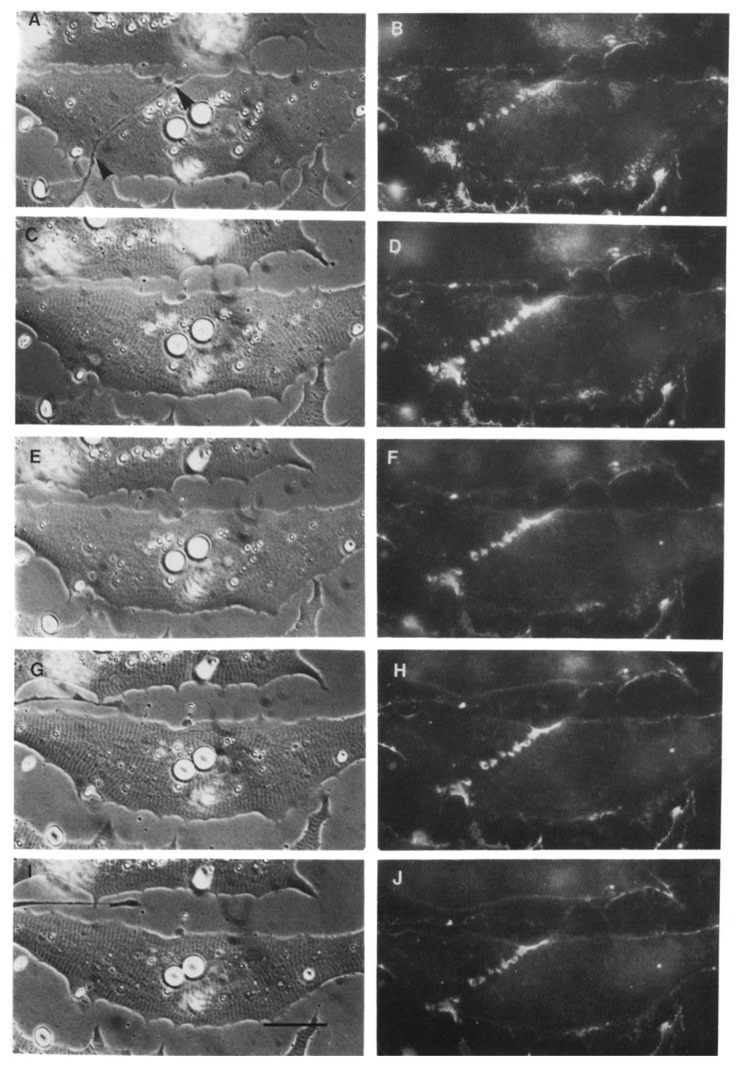
Stability of junctional HSPG deposits after denervation. The *Xenopus* nerve-muscle culture was exposed to continued presence of a fluorescently-labeled monoclonal antibody, and photographed at daily intervals. The nerve (arrowheads in A) disappeared between the first (**A**,**B**) and second day (**C**,**D**) in culture. HSPG organization increased along the path of nerve-muscle contact between the first and second observation (**C**,**D**) and remained static thereafter (**F**,**H**,**J**) despite loss of the nerve (**C**,**E**,**G**). The intensity of HSPG staining decreased in panels **F**, **H**, and **J** owing to fluorochrome bleaching in response to the repeated excitation. Scale: 40 µm. Reproduced with permission from Anderson et al., J. Cell Biol. 1984;99:1769-84. [97]

**Figure 5:**
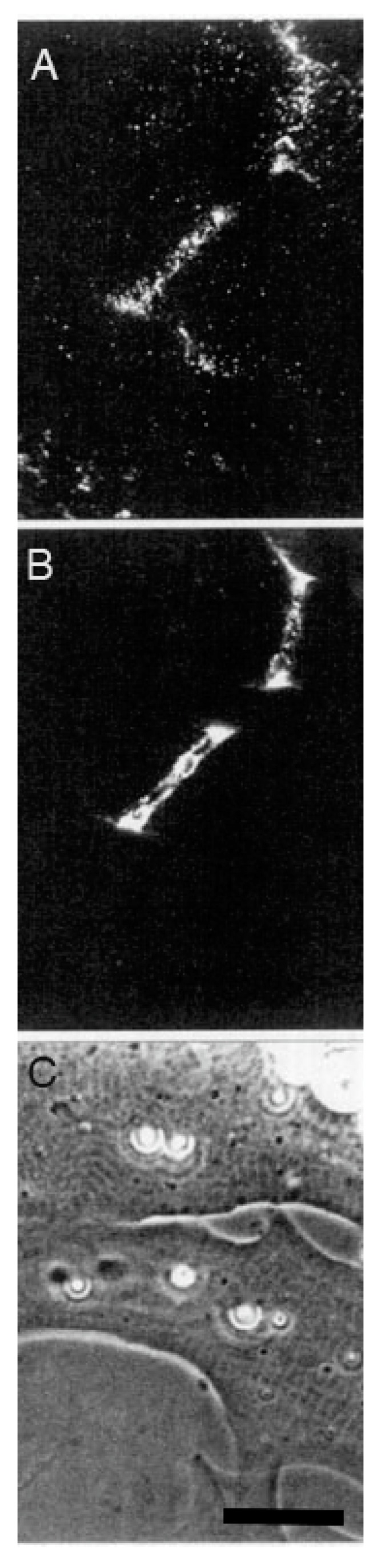
Survival of synaptic agrin and AChR accumulations after nerve degeneration in an older *Xenopus* nerve-muscle culture. Agrin was stained with a monoclonal antibody (**A**) and AChRs were labeled with α-bungarotoxin (**B**), using contrasting fluorochromes. Note the intense AChR staining across the center of the two myocytes (**B**), and the co-localized agrin deposits (**A**). The nerve is no longer visible in the phase contrast reference image (**C**). In this figure, where the nerve has persisted long enough for agrin deposits to be laid down, synaptic AChR aggregates have remained. In contrast, synaptic AChR aggregates disperse if denervation occurs within a day after synaptic differentiation becomes evident. Scale: 20 µm. Adapted with permission from Anderson et al., Dev. Biol. 1995;170:1-20. [112]

The denervation-induced dispersal of synaptic AChR clusters was not paralleled by corresponding changes in the basal lamina however; as shown in Figure 4, synaptic HSPG deposits became more pronounced between day 1 and day 2, and after denervation remained essentially unchanged for several days thereafter [97]. As the remodeled basal lamina was clearly not able on its own to prevent AChR dispersal, the question arises as to what agent or process led to the apparent increase in stability of synaptic AChRs in older contacts. Given the evidence that agrin makes a delayed appearance (Figure 3), it is an obvious candidate. This suggestion is further supported by evidence that once deposited, agrin can stabilize AChR clusters, as shown in Figure 5. In this older culture the nerve has died, but the typical fluorescence profile of a late-stage culture is evident, in that there are advanced synaptic AChR aggregates, together with co-localized deposition of agrin [112]. It is tempting to conclude that the appearance of agrin, relatively late in the overall process (Figure 3), has stabilized the synaptic AChR clusters so that they no longer depend on the presence of an intact nerve.

At first glance, these findings suggest that different mechanisms for the maintenance of synaptic AChR clusters may be involved in the amphibian cultures, as compared to those operative in the mammalian system [37,38]. The amphibian synaptic AChR aggregates do not require any neuronal stimulus such as ACh to mediate their dispersal; the nerve, rather than acting to disperse them, clearly acts to maintain them. This difference between the two experimental systems may be more apparent than real, however, if, as suggested in Section 3.1, synaptic AChRs at mammalian synapses are actually re-aligned receptors, newly aggregated under the nerve, having moved there from their positions in the prepattern. If so, they too may be unstable at the earliest stages of their re-alignment.

Such a mechanism would be supported if future studies determine that mammalian postsynaptic differentiation also involves a proteolysis-driven remodeling of the basal lamina, similar to that of the amphibian culture system. Since, as discussed above, laminin is first removed and then redeposited during this process, we would expect a similarly late appearance of agrin, given its dependence on laminin for anchoring in the basal lamina. Conversely, if no such remodeling occurs, it would strengthen the case that the AChRs in the prepattern are simply co-opted directly, in accordance with the model of Sanes and Yamagata [60] and Burden [61].

##### The Amphibian Culture System: Conclusions, Caveats, and Recommendations

Taken together, several lines of evidence all incline toward the conclusion that neuronal agrin appears too late to act as an inducer of postsynaptic differentiation in the amphibian cultures, but that once deposited it acts to stabilize synapses that are already relatively advanced in their formation. The loss of this stabilizing function would be more than sufficient to account for the devastating impacts of the early agrin knockouts [23,24]. As with the synapses of the transgenic described by Kim and Burden [47]; (see Section 3.2.2 above), these data are most consistent with an inductive process that does not involve either agrin or prepatterning.

At present these studies in the amphibian system stand as the only attempts to assess the sequence of events such as basal lamina remodeling and agrin deposition during the development of the NMJ. Several caveats must be acknowledged respecting these observations, however, in particular regarding the key finding that agrin appears to come in too late to be an inducer. First, while Anderson et al. [112] went to considerable lengths to control for penetration artifacts that might lead to inconsistent staining of agrin deposits, technical difficulties, whether reflecting issues of penetration or local proteolytic action on specific epitopes, can never be excluded definitively. For example, focal proteolysis of matrix constituents is a constant feature attending synaptogenesis in the amphibian culture system [14,115] and agrin is known to be subject to hydrolysis by several metalloproteinases [116,117], as well as by neurotrypsin, a serine proteinase [118,119]. More generally, in a context where staining is, in the words of Anderson et al., “erratic” [112], definitive conclusions will require a complete description that can account for the presence of agrin at some synapses and its absence in others.

A second issue derives from the detailed molecular analyses of agrin’s interactions, specifically the need for laminin to be present in order for the LN-agrin isoforms to bind to the extracellular matrix. Although these findings of Burgess et al. [100] reinforce the longstanding view that agrin acts physiologically as a constituent of the remodeled basal lamina, implying a late appearance of agrin in the amphibian culture system, they do not in themselves definitively rule out an inductive role in postsynaptic differentiation. In principle, there remains an early temporal window in which agrin could still mediate the initial inductive interaction, one that precedes its incorporation into the basal lamina, and initiate the entirety of the succeeding developmental sequence; ultimately, this would even include further accumulation of the LN-agrin isoform through its binding to laminin. While this scenario seems highly unlikely, especially in view of the in vivo knockouts where synapse formation persists in agrin’s absence [37,38,47,84], it remains a formal possibility.

These considerations require a re-examination of the exact sequence of events that follow contact between nerve and muscle, in particular whether the deposition of neuronal agrin precedes or follows basal lamina remodeling. Given the technical constraints operative a generation ago, the necessary repeated observations of a given synaptic contact were difficult to perform. Modern approaches, however, include the use GFP-modified constructs that obviate concerns regarding the use of staining reagents, as well as advances in the sensitivity of fluorescence microscopy that permit examining multiple channels while minimizing fluorochrome bleaching [120]. This should facilitate repeated assessments of several key molecular actors at all stages of synapse formation, not only in studies with the amphibian culture system, but ultimately with in vivo preparations, the gold standard for any proposed mechanism.

## 4. Synapse Induction: Alternatives to Agrin

If deposition of neuronal agrin by motor neurons is not the mechanism for conferring specificity and inducing postsynaptic differentiation, the question arises as to what an alternative process might be. At the level of the nervous system as a whole, numerous classes of mechanisms have been defined for ensuring appropriate connections between neurons and their targets, including such processes as inhibitory interactions between surface molecules, placeholder and guidepost cells to direct axon growth, and actions to eliminate inappropriate synapses [60,121]. Many of these are not relevant for the present discussion, however, which will focus on the simplest of cases, that of nerve on undifferentiated muscle, as described both in vivo and in vitro (see Section 2.1). The logic in so doing is not that the answers will necessarily provide a definitive conclusion for all nerve-muscle synapses; rather, it is that the simplest case does require explanation for itself, and that it is likely that mechanisms so revealed will provide insights into the wider spectrum of NMJs. This review will examine four potential mechanisms, namely ligand receptor interactions, Wnt signaling, focal pericellular proteolysis, and integrin signaling. All four have either been examined in other contexts in the nervous system, with potential relevance to NMJ formation, or have been studied directly at the NMJ.

### 4.1. Ligand Receptor Interactions

Based on work done elsewhere in the nervous system, there is in principle a plethora of potential candidates for mediating nerve-muscle interaction through homophilic or heterophilic binding, including such families as the cadherins, protocadherins, neurexins and neuroligins, ephrins and eph kinases, semaphorins and neuropilins or plexins, and members of the immunoglobulin family (for reviews see [60,121,122]). One example that has been examined is neural cell adhesion molecule (NCAM), for which different isoforms were found to mediate stabilized contacts between growth cones and cultured myotubes [123]; however, complete ablation of NCAM does not prevent formation of NMJs, although they are somewhat reduced in size and manifest some physiological deficits [124,125]. Other families have not been subject to close examination as candidates for initiating postsynaptic differentiation, however, for the simple reason that the concept of a ligand-receptor interaction was dominated for so long by the focus on agrin. As discussed above, however, there are reasons to believe that this focus is misplaced, as agrin’s binding to Lrp4 is more likely to serve as a stabilizing rather than an inducing function, thus leaving the situation open for consideration of other possibilities.

At present, therefore, there are no positive data to discuss regarding alternative ligand-receptor pairings, but there are at least two candidates of potential interest that can be ruled out, namely the amyloid precursor protein, together with its two related amyloid precursor-like proteins, and presynaptic Lrp4. In both cases gene knockout data argue against their involvement in the initial inductive process. Regarding the amyloid precursor and the two precursor-like proteins, several studies have implicated them as being essential for proper synapse formation [126,127], reflecting molecular interactions with Lrp4 and agrin [128]. Nonetheless, simultaneous ablation of all three proteins, while leading to perinatal lethality, still leaves the mice able to breathe for many hours or even days [129], so that, as argued by Caldwell et al. [130], the initial steps involved in NMJ formation must already have taken place without them.

With respect to neuronally-derived Lrp4, an elegant study, involving the ablation of Lrp4 in nerve, muscle or both, demonstrated that soluble and active fragments of presynaptic Lrp4 are generated through local proteolysis [65]. In the absence of postsynaptic Lrp4, these fragments mediate the formation of AChR clusters, although the latter are significantly reduced in size relative to the normal case. Interestingly, in the context of overall postsynaptic abnormality, they are still sufficient to prevent neonatal mortality, with the mice surviving for as long as a year. However, in this paper the authors also demonstrated that synapse formation proceeds normally when only neuronal Lrp4 is ablated, implying that the presence of presynaptic Lrp4 fragments is irrelevant in the presence of an intact postsynaptic complement of Lrp4, one that swamps out any neuronal contribution. In all other studies this condition is met, including those involving synapse induction in the absence of agrin (see Section 2.2), meaning that it is unlikely that neuronally-derived Lrp4 provides an inductive signal.

### 4.2. Wnt Signaling

The relevance of Wnt signaling to NMJ formation has been the subject of numerous studies, beginning with the finding that Dishevelled, a protein implicated in Wnt-mediated pathways, interacts with MuSK to regulate AChR clustering [131]; for reviews, see [75,132]. Detailed analysis of the underlying processes has proved difficult, however, owing to the sheer number of Wnt proteins, numbering some 19 in mammals, with yet more in other vertebrate and invertebrate species [132]. Moreover, Wnt signaling is especially complex, involving as it does the canonical pathway leading to regulation of gene transcription, as well as the two noncanonical pathways affecting the cytoskeleton (planar cell polarity pathway) and intracellular calcium metabolism [133]. This complexity has been reflected in work on the NMJ, as studies using in vivo and in vitro protocols have variously reported that some Wnts can promote formation of AChR clusters, while other Wnts are inhibitory; see [75] for a recent review.

With respect to the particular focus of the present review, namely the nature of the inductive interaction when nerve and muscle meet, studies in both mice and zebrafish have suggested that Wnt signaling does not play an essential role. As noted in Section 3.1, experiments in zebrafish, involving transgenic manipulation of *unplugged* (the zebrafish version of MuSK), as well as morpholino-mediated knockdown of Wnt11r, which binds to *unplugged*, demonstrated that interruption of Wnt signaling leads to abolition of AChR prepatterning and impaired axonal targeting. However, these perturbations did not prevent the formation of functioning NMJs, demonstrating not only that synapse induction had occurred, but also that prepatterning is not essential for synapse formation in this system [76]. Furthermore, in a recent study employing mutation of the Wnt ligand secretion mediator (*Wls*) gene, a strategy aimed at abolition of signaling by a multitude of mammalian Wnts, Shen et al. [134] found that ablation of Wls function in muscle or Schwann cells did not result in any significant phenotype. In contrast, mutation in motoneurons led to both presynaptic and postsynaptic abnormalities, but once again NMJs did form, and the mutant mice, while exhibiting muscle weakness and reduced growth after birth, still survived. Given that some Wnts may not be dependent on Wls, these results cannot definitively rule out Wnt-mediated signaling as a key inductive process, but they strongly suggest that, as with zebrafish, some other mechanism is involved.

Very similar results were reported by Remédio et al. [77] who found that conditional deletion of *Wls* did not prevent synapses from forming in mice, nor did ablation of the cysteine-rich-domain (CRD) of MuSK that is required for Wnt binding. Thus, Wnt signaling was dispensable for synapse formation, and was not even required for generation of the AChR prepattern. Although another group, using mice with a different genetic background, reported that deletion of the MuSK CRD did lead to profound deficits in prepatterning and synapse morphology, functional synapses were again induced, inasmuch as these mice also survived [135]. Taken together, the studies from both mice and zebrafish reinforce the conclusion that Wnt signaling, like the amyloid precursor family, may be important for synapse maturation and maintenance, but is unlikely to be essential for the induction of postsynaptic differentiation. Accordingly, in a recent review of the relationship of Wnt signaling to synapse formation at the NMJ, Li et al. [75] argued that its role is essentially modulatory, in contrast to the central role played by agrin. On present evidence, this conclusion seems justified.

### 4.3. Focal Pericellular Proteolysis

As discussed in Section 3.2.2, synapse formation in the amphibian in vitro system is invariably associated with a removal of pre-existing basal lamina constituents and their ultimate re-deposition, at highly elevated concentrations, along the path of nerve-muscle contact (see Figure 1 and Figure 2). The initial removal satisfies two key requisites for an inductive process, time of occurrence and specificity with respect to cell type. In cultures with a mixed neuronal population, it occurs only at nerve-muscle contacts that go on to make synapses, and synapses only form at contacts where this removal is observed [14]. Moreover, the removal of HSPG and laminin occurs early, and it is the first indication that a synapse will form.

The most reasonable interpretation of these observations is that the removal of laminin and the basal lamina HSPG reflects a local proteolysis that is specific to contacts between muscle and motor neurons; this process is clearly regulated, inasmuch as the same constituents that were removed are subsequently laid down at high concentration. As is evident from Figure 1, the width of the zone of depleted basal lamina constituents is approximately five times wider than the diameter of the motor neuron, a typical finding in this system [14,107,115]. In contrast, the zone of removal of fluorescent substrates adsorbed to the cover glass is only as wide as the nerve itself [115], suggesting that the wider zone of proteolysis of laminin and HSPG in the synaptic cleft reflects not just the action of neuronal proteinases, but activation of muscle proteinases as well.

The essential issue, however, is to establish that the apparent proteolysis serves a functional role. It is possible that motor neurons in these cultures are inherently more proteolytically active than other nerve types, and that basal lamina removal, while correlated with subsequent synapse formation, reflects no causal link whatever. In this case, the apparent regulation of this activity, as evidenced by the ultimate re-deposition of laminin and HSPG at high concentration in the synaptic cleft, would have nothing to do with regulation of cell-cell signaling; instead, it would simply be a necessary shutting down of local extracellular proteinase activity in order that a specialized synaptic basal lamina may form. In principle, this question can be approached through the use of proteinase inhibitors of varying specificity, both naturally occurring and synthetic [136,137,138], to assess not only whether synapse formation is affected, but also, in the event of blockage, to determine the time point at which the inhibition is functionally relevant.

#### 4.3.1. Proteinases as Modulators of Nervous System Processes

If focal proteolysis is a key mechanism in mediating cell-cell interaction at the NMJ, this would hardly be a novel phenomenon for the nervous system. The involvement of proteinases has been extensively reviewed, owing to their importance for a multitude of physiological and pathological processes [139,140]; these include long term potentiation [141,142,143], growth cone interactions [144], hippocampal cell differentiation [145], amyloid clearance [146,147], and axonal regeneration [148]. The two principle classes of proteinases involved in these processes are the metalloproteinases, including the matrix metalloproteinases (MMPs) and the ADAM (a disintegrin and metalloproteinase) family [140], and serine proteinases such as neurotrypsin and the tissue plasminogen activator/plasmin system [136,139].

Several factors contribute to the capability of proteinases to mediate the extraordinary variety of interactions that are required for establishing specific synaptic connections. First, there is a plethora of potential mechanisms by which pericellular proteolysis may contribute to synapse induction. Local proteolysis can modulate the activity of kinase signaling networks, cell surface receptors, and agonists such as chemokines, cytokines and growth factors; depending on the proteinase and target protein, these can take the form of activating or degrading cleavages [149,150,151,152]. There is even an entire family, the proteinase-activated receptors, which explicitly respond to proteolytic removal of an N-terminal blocking sequence [153].

This complexity extends to the range of action of individual enzymes. A recent tabulation of reported MMP substrates ranged as high as 94 for MMP2, with 10 members of the MMP family having 39 substrates or more [154], while a proteomic analysis of the neuronal ablation of another key metalloproteinase, ADAM10, identified 91 candidate substrates [155], in addition to several dozen already tabulated in earlier reviews [156,157]. Secondly, many proteinases are secreted as inactive zymogens, and are activated only upon cleavage by another proteinase, either of the same or a different class [158,159], with reactions further modulated through the action of endogenous inhibitors [136,137]. This allows for precise specificity in generating a final product, as exemplified by the clotting system [160]. Moreover, proteinases derived from different tissues can cooperate to effect tissue remodeling [161], to the point where proteinases from different tissues can participate in a single cascade [162]. Thus an interaction between nerve and muscle surface proteinases to generate basal lamina remodeling, as proposed by Champaneria et al. [115], is well within established phenomenologies.

#### 4.3.2. Proteolysis as Mediator of Synapse Maintenance

Finally, it should be noted that even if focal proteolysis ultimately proves not to be important for the induction of a postsynaptic apparatus, it may still play another key role, as it is well established that the activity of MMPs can mediate signaling through the release of growth factors [163]. At the NMJ, it is known that a number of growth factors are bound to the heparan sulfate side chains of the basal lamina HSPG, and it is likely that their local release is a significant contributor to long-term motor neuron maintenance. The first evidence for this mechanism was provided by Fischbach and colleagues, supporting a model for localized proteolytic release of neuregulin-1 from the basal lamina [164]; since then a number of proteins have been shown to promote motor neuron survival in the context of diseases such as amyotrophic lateral sclerosis, and, like neuregulin-1 [165], to possess heparin-binding domains. These supporting proteins could be produced by nerve, glia, or muscle, and their lack could be felt either directly by the nerve or indirectly by modulating the activity of the enveloping Schwann cell. They include vascular endothelial growth factors A [166] and B [167,168], hepatocyte growth factor [169,170], pleiotrophin [171,172], glial cell-line-derived neurotrophic factor [173,174], bone morphogenic protein 4 [175,176], and, through the intermediation of their dedicated binding proteins, insulin growth factors 1 and 2 [177,178,179,180,181]. In addition, several members of the FGF family, another group known to react with glycosaminoglycan side chains [170], have been shown to promote presynaptic differentiation [182]. Thus, it is likely that the findings regarding the local release of neuregulin-1 apply to these other growth factors as well.

Local proteolytic release of growth factors could even contribute to the initial inductive process, but at present there is no evidence that any of these factors acts at this level. In contrast, they have all been shown to enhance motor neuron survival or differentiation. In principle, therefore, aberrant regulation of their release could be important in pathological conditions where a connection to proteolysis has been identified; for example, it would be of interest to examine whether growth factor availability is linked to the involvement of MMP9 in the etiology of motor neuron disease [183].

In summary, depending on the outcome of further researches into synapse induction, we are thus left with the distinct possibility that focal proteolysis, advanced herein as an alternative to agrin as an inductive process, may instead, like agrin, turn out to be a contributor to the stability of synaptic connections, while the key inductive interaction remains to be found elsewhere.

### 4.4. Integrin Signaling

Integrins are membrane spanning heterodimers, composed of non-covalently linked α and β subunits, that act as adhesion receptors to mediate signaling through interactions with extracellular matrix ligands such as fibronectin and laminin [184,185,186]. This signaling is essential for proper synaptic function in several loci of the nervous system [187]. In the hippocampus, dendritic spine enlargement and remodeling is mediated through β1integrin signaling that in turn is dependent on the activity of the matrix metalloproteinase MMP9 [141,188], while at the NMJ blockage of β1integrin function in the early postnatal period or following injury leads to motor neuron death [189]. In addition, studies with cultured myotubes demonstrated that β1integrins co-localize with AChR clusters, and are essential for their formation in response to bath application of agrin and laminin [190,191,192].

**Figure 6:**
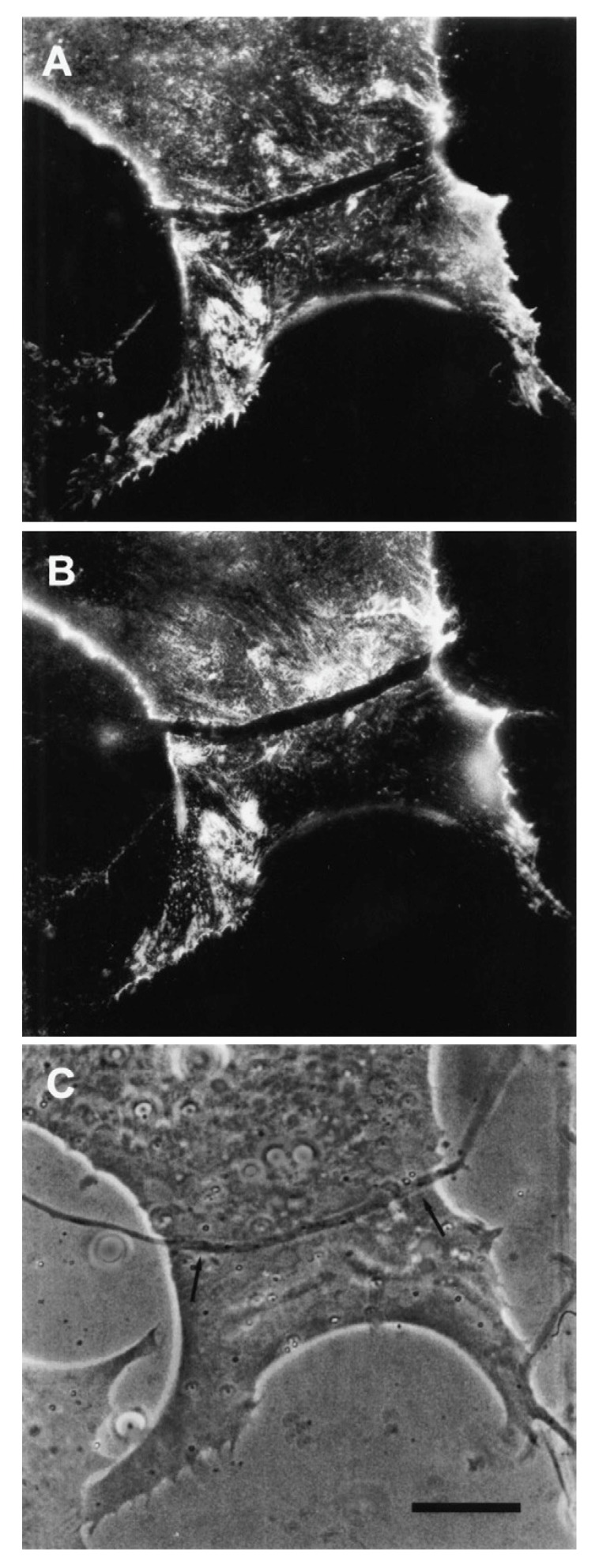
Elimination of β1 integrin and laminin accumulations before the onset of postsynaptic differentiation. Integrin (**A**) and laminin (**B**) were stained with antibodies conjugated with contrasting fluorochromes, prior to nerve addition. The cultures were examined before the onset of synaptic differentiation. Note that integrin (**A**) and laminin (**B**) accumulations have both been eliminated along the entire path of nerve-muscle contact, shown by the arrows in the phase contrast reference image (**C**). Scale: 20 µm. Reproduced with permission from Anderson et al., Mech. Dev. 1997;67:125-39. [193]

**Figure 7:**
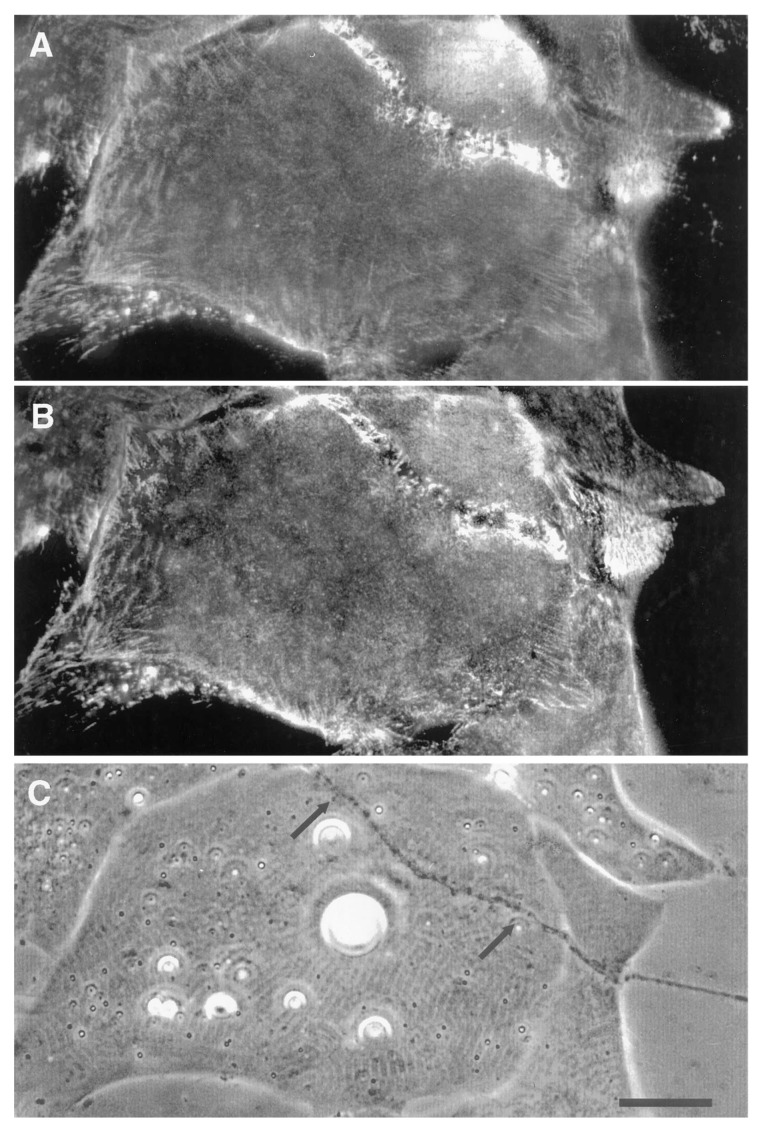
Aggregation of β1 integrin in the muscle membrane matches that of laminin in the basal lamina. In this older *Xenopus* nerve-muscle co-culture, laminin (**A**) and β1 integrin (**B**), are stained with monoclonal antibodies conjugated to contrasting fluorochromes. The distribution of the two antigens is essentially congruent, both along the path of this well-developed synaptic contact, shown in (**C**), and over the remainder of the muscle cell as well. Scale: 20 µm. Reproduced with permission from Anderson et al., Mol. Cell. Biol. 1996;16:4972-84. [107]

Using the amphibian culture system, Anderson et al. [107,193] found that the early removal and subsequent re-deposition of basal lamina constituents is accompanied by re-organization of postsynaptic β1integrin. First, it is removed (Figure 6) and ultimately it is concentrated at the synapse, concomitant with the accumulation of laminin in the basal lamina (Figure 7). This synaptic accumulation of integrin resembles that of AChRs, with the exception that, unlike the situation with AChRs, integrin is also found over the myocyte surface (compare Figure 2 and Figure 7). The synaptic accumulation is not surprising, since laminin is a key basal lamina ligand for integrin [186]. Thus, correlations between the two molecules may simply represent the attachment of the β1integrin to its basal lamina binding partner, without there being any direct relation between integrin signaling and subsequent synaptogenesis; this linkage may also lead to the aberrant basal lamina deposition that has been reported in mice lacking the α3 integrin subunit [194].

However, the possibility remains that the impacts of innervation on integrin distribution are causative of later events in synaptogenesis, specifically AChR clustering. If so, the use of integrin inhibitors [195,196] may shed further light on the underlying molecular interactions with key actors such as agrin, Lrp4, and MuSK. In this connection, it is of particular interest that while genetic ablation of β1integrin in motor neurons has little effect, loss of the same gene in muscle abolishes nerve termination and prevents formation of the NMJ in mice, with consequent lethality [197]. Of particular note, the lethality is embryonic, in contrast to the perinatal death that results from ablating all three amyloid precursor and precursor-like proteins (see Section 4.1), meaning that, at the very least, integrin signaling remains a candidate for involvement in the key inductive interactions occurring between nerve and muscle at their first contact.

Finally, a recent report from a completely unrelated field of cell biology, the etiology of hepatocellular carcinoma, presents an intriguing possibility for a unified mechanism for synaptogenesis involving integrin, agrin and Lrp4-MuSK signaling. Agrin is overexpressed in hepatocellular carcinoma and promotes cellular proliferation [198]; further detailed analysis of this interaction has revealed that agrin acts as a bridge between both the integrin-linked kinase and Lrp4-MuSK pathways, transducing changes in extracellular matrix rigidity and ultimately resulting in the activation of the nuclear transcription factor YAP [199,200]; see commentary of Xiong and Mei [201]. YAP in turn has been implicated as a downstream regulator of NMJ formation [202]. Recently, a similar mechanism, involving agrin-mediated interactions with β1 integrin, Lrp4, and MuSK, has been shown to facilitate adhesion between cancer cells and endothelial cells, concomitant with enhancing the stability of another key cellular constituent, vascular endothelial growth factor receptor-2; together, these interactions lead to the promotion of tumor angiogenesis and metastasis [203]. While much remains to be elucidated as to the molecular details, the overall picture is consistent with the conclusions of Section 3, that agrin acts as a stabilizing factor. A possible model would thus involve an initial proteolysis-mediated remodeling of the extracellular matrix and concomitant integrin distribution, leading to signaling through integrin-mediated pathways. Agrin would act not only to potentiate these but, similar to events in liver cancer, also serve as a bridge to activate Lrp4-MuSK signaling that further stabilizes the nascent synapse.

## 5. Conclusions

In their “paradigm lost” analysis, Kummer et al. refer to the vertebrate skeletal neuromuscular junction, a reference both specific, ruling out heart and smooth muscle but including twitch and tonic, as well as general, meant to apply throughout the Vertebrata [44]. This is the traditional view, one that emphasized evolutionary parsimony at the molecular level, and that underlay the elaboration of the agrin hypothesis, recently modified to include both inductive and stabilizing functions for agrin. The principal conclusion of the present review is that there is reason to question the relevance of the inductive action of agrin. This conclusion is derived primarily from in vivo studies, showing that NMJs form in mice lacking the agrin gene [37,38,47,84], as well as from in vitro data, suggesting that agrin’s deposition occurs relatively late, rather than early, during synapse formation [107,112]. The clear implication is that another molecular mechanism, as yet undefined, begins the process of postsynaptic differentiation. Several candidates for this role have been advanced herein. Only additional studies can confirm or refute the involvement of any of these proposed mechanisms, and establish whether or not synapse induction involves a fundamental molecular process that operates at every vertebrate skeletal nerve-muscle interface. Irrespective of the answer, it is safe to assume that these findings will be relevant not only to the NMJ, but also to cell-cell interaction elsewhere in the nervous system.
